# Prevalence and associated factors for HIV, HBV and syphilis coinfections among pregnant women attending antenatal care in Tanzania

**DOI:** 10.1371/journal.pone.0329068

**Published:** 2025-08-01

**Authors:** Agnes Fridomu Njau, Masanja Robert, Anath Rwebembera, Renatus Kisendi, Chacha Maro, Grace Dennis, Mukome Nyamhagatta, Michael Msangi

**Affiliations:** 1 Tanzania Field Epidemiology and Laboratory Training Program, Dar es Salaam, Tanzania; 2 Department of Epidemiology and Biostatistics, Muhimbili University of Health and Allied Sciences, Dar es Salaam, Tanzania; 3 Ministry of Health, Dodoma, Tanzania; Centers for Disease Control and Prevention, UNITED STATES OF AMERICA

## Abstract

**Background:**

Coinfection with HIV, hepatitis B virus (HBV) and syphilis increases the risk of vertical transmission. Hence, affecting overall maternal health and child health outcomes. The Tanzanian government is planning to add HBV screening to the existing Prevention of Mother to Child Transmission (PMTCT) of HIV and syphilis program; however, the burden of coinfections in the country is unknown. Therefore, this study aimed to determine the prevalence of HIV, HBV and syphilis coinfections and their associated factors among pregnant women receiving antenatal care in Tanzania.

**Methods:**

A facility-based cross-sectional study design was conducted, utilizing data from the national feasibility study of triple testing for HIV, syphilis and HBV among pregnant women. The data were analysed via STATA version 16.1, and bivariate and multivariate logistic regressions were used to check for associations. Variables with a P value of < 0.05 were considered statistically significant.

**Results:**

A total of 7,828 pregnant women were enrolled, 0.4% (95% CI 0.3–0.6) of whom were coinfected. The prevalence rates for HIV/HBV, HIV/syphilis, HBV/syphilis and HIV/HBV/syphilis coinfections were 0.1% (95% CI 0.1–0.2), 0.2% (95% CI 0.1–0.4), 0.1% (95% CI 0.0–0.2) and 0.0% (95% CI 0.0–0.1), respectively. History of multiple sexual partners (AOR 6.1; 95% CI: 1.3–29.7, P = 0.025) was associated with HIV/HBV coinfection. Age 25–49 years (AOR 13.5; 95% CI 1.8–103.8, P = 0.012) and marital status (AOR 0.2; 95% CI 0.1–0.8, P = 0.018) were associated with HIV/syphilis coinfection. For HBV/syphilis coinfection, marital status (AOR 0.1; 95% CI 0.0–0.9, P = 0.036) and history of multiple sexual partners (AOR 16.8; 95% CI 2.5–114.9, P = 0.004) were independently associated.

**Conclusion:**

Coinfections are present among pregnant women in Tanzania; therefore, it is important to include hepatitis B screening in the existing PMTCT of HIV and syphilis program. Interventions should focus on single, child-bearing women with multiple sexual partners.

## Introduction

HIV (human immunodeficiency virus) is a virus that attacks the body’s immune system [1]. Globally, approximately 39.9 million people are living with HIV. In sub-Saharan Africa, approximately 20.8 million people are living with HIV [2], and in Tanzania, approximately 1.7 million people are living with HIV [3], with the prevalence of HIV among pregnant women being 5.9% [4]. It is estimated that in the absence of interventions, the rate of vertical transmission of HIV ranges from approximately 15–45% [5].

Hepatitis B virus (HBV) is a virus that infects the liver and can present as an acute or chronic infection [6]. Approximately 3.3% of the world’s population is infected with HBV, with approximately 1.2 million new infections and 1.1 million deaths in 2022 [6]. In Tanzania, 4.7% of pregnant women are infected with HBV [7]. In the absence of intervention, the rate of vertical transmission of HBV ranges from 70% to 90% (HBeAg-positive) and from 10% to 40% for HBeAg-negative patients [8].

Syphilis is a sexually transmitted disease that can easily be manageable and curable. There were approximately 8 million syphilis-infected adults in 2022 [9]. In Tanzania, the prevalence of syphilis among pregnant women is 1.4% [10]. Untreated maternal syphilis can lead to stillbirth, preterm or low birth weight, and fetal or neonatal death in 50–80% of cases [9]. These infections pose a significant public health concern, as they affect overall maternal health, fetal development and child health outcomes.

Coinfection with HIV, hepatitis B or syphilis refers to infection with at least two of these diseases, which can significantly increase the risk of vertical transmission from mother to child. The global prevalence of HIV/HBV coinfection is 7.4% [6]; in sub-Saharan Africa, it is 3.3% [11]; in Rwanda, it is 4.1% [12]; in Angola, it is 6.3% [13]; in Ethiopia, it is 2% [14] and 10.5% in Tanzania [15]. This coinfection is associated with poor fetal outcomes, including low birth weight and incident HIV infection [16].

The rate of HIV/syphilis coinfection is 1.3% in Ethiopia [14], 0.7% in the Republic of Congo [17], 5% in Angola [18] and 37.7% in Botswana [19], and it is associated with adverse fetal outcomes, namely, still birth and low birth weight [19]. The prevalence of hepatitis B virus (HBV)/syphilis coinfection is 0.8% in Ethiopia [14]. Owing to the high burden of these diseases and coinfections globally, the WHO and member countries are committed to eliminating the vertical transmission of these three diseases (triple elimination) [20].

In Tanzania, the government is planning to add HBV screening to pregnant women under the Prevention of Mother to Child Transmission of HIV and syphilis program, with the aim of eliminating vertical transmission of HIV, HBV and syphilis, but little is known about the burden of these coinfections. Hence, a study was conducted to determine the burden of coinfections among pregnant women, which has not been explored previously, and to further assess the factors associated with coinfections among pregnant women. These findings will help the country implement triple elimination of HIV, HBV and syphilis.

## Materials and methods

### Study design and study setting

We conducted a facility-based cross-sectional study design, utilizing data from the national feasibility study of triple testing for HIV, syphilis and HBV among pregnant women receiving antenatal care in four regions (Dar es Salaam, Geita, Mbeya and Njombe) with the highest prevalence rates of HIV, HBV and syphilis, from October 2023 to June 2024. A total of 17 district councils and 102 health facilities were involved in these regions.

### Data collection

We retrieved screening and baseline data from the feasibility of triple testing on the Kobo Toolbox of the PMTCT program under Ministry of health on 24^th^ September 2024 and exported it to Microsoft Excel. The screening data, which included the sociodemographic characteristics of our study participants and their consent for triple testing, while baseline data, included triple testing results of our study participants, and their risk factors for coinfections. The two datasets were merged together using unique identifiers generated from district council names, health facility names and participant identification number. The variables included in our study were age, place of residence, marital status, level of education, employment status, level of health facility, and risk behaviours such as having multiple sexual partners, blood transfusion history, tooth extraction history, history of ear piercing, condom use, operation history, incarceration history, and sharing of needles and syringes and family members with HBV.

### Laboratory testing methods

The triple testing results used in this study were obtained from the implementation of the feasibility project of triple testing under the national PMTCT program, following standardized national guidelines, with all testing conducted by healthcare providers. HIV and syphilis screening were performed using HIV/syphilis dual testing kits approved for use across all regions. In cases where dual kits were unavailable, separate HIV antibody rapid tests and syphilis rapid diagnostic tests were used. Hepatitis B surface antigen testing was performed using kits recommended by the national program.

### Determination of infection status

As this study used secondary data, infection status was determined based on documented results. For HIV, women attending their first ANC visit were tested at the facility and results were recorded directly. For those who had been tested previously in pregnancy, HIV results were obtained from their ANC cards. For women in the third trimester who had undergone repeat HIV testing, updated results were also obtained from ANC cards. For syphilis and hepatitis B, testing was done once. Results for these infections were either recorded during the visit or extracted from ANC cards if testing had been done previously. Based on this documentation, the final HIV, syphilis, and hepatitis B status of each woman was classified as either positive or negative.

### Data extraction and interpretation

**Fig 1:**
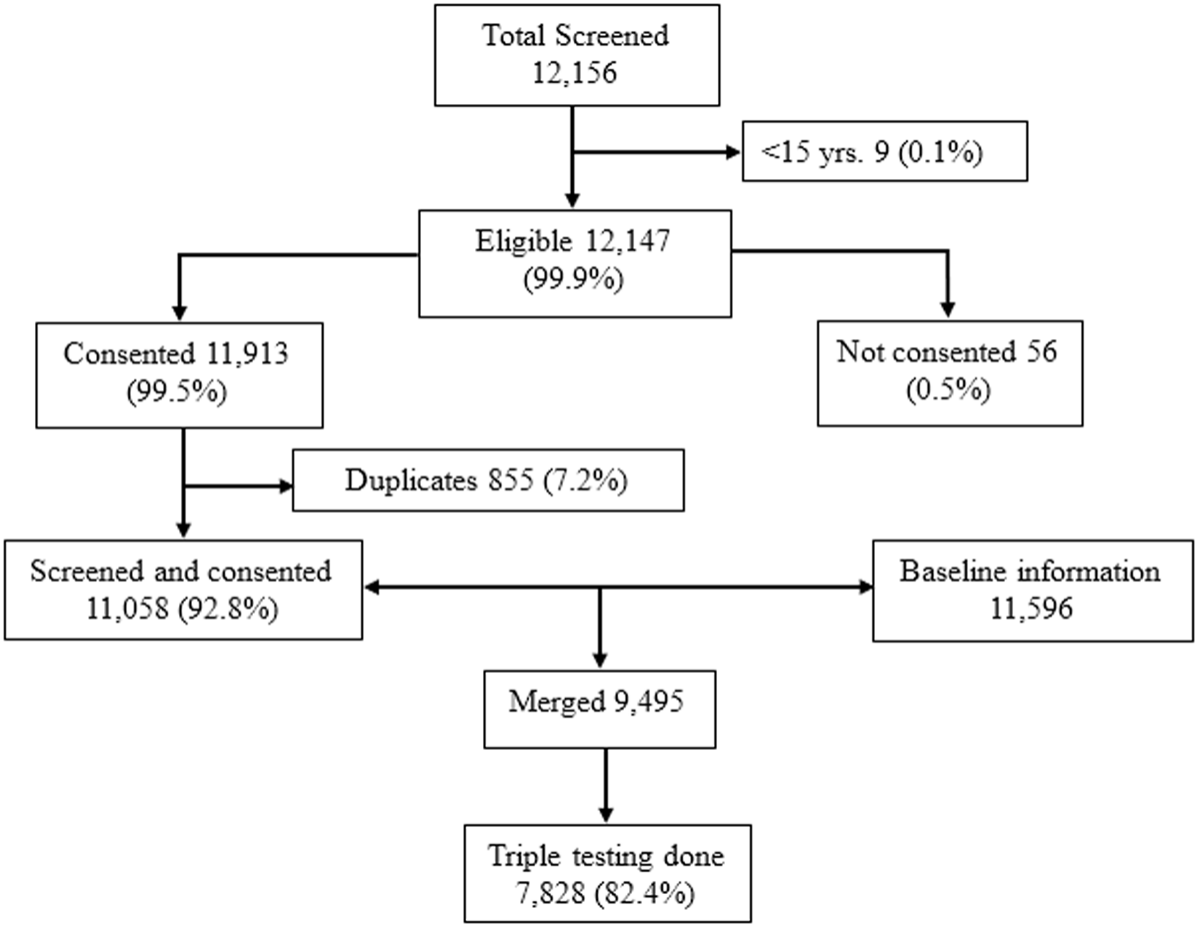
Flowchart of the final dataset analysed.

We extracted data from a total of 12,156 pregnant women who were screened for triple testing. Of these, 12,147 (99.9%) were ≥ 15 years. In addition, 11,913 (99.5%) participants consented to participate in the study. After removing duplicates from the screening dataset, 11,058 (92.8%) pregnant women who were screened and consented to participate were included. Furthermore, we extracted baseline data from 11,596 pregnant women who were merged with the 11,058 pregnant women screened for triple testing. After merging, only 9,455 data points had both screening and baseline information, and from the merged data, 7,828 (82.4%) pregnant women who had received all three tests for HIV, HBV and syphilis were included in our final dataset (Fig. 1).

### Data analysis

The data were cleaned and analysed with STATA version 16.1. Frequencies and proportions were used to summarize the sociodemographic characteristics of the study participants, and age was summarized as the median and interquartile range. The prevalence of coinfections among pregnant women were calculated, with the respective 95% CI. The measure of association was calculated via the odds ratio, and a 95% confidence interval was used. Bivariable logistic regression was used to determine whether there was an association between the independent and dependent variables and their respective P values. Variables with a P value of < 0.2 from the bivariable model were entered into the multivariable analysis to control for confounders. A P value of < 0.05 was considered statistically significant in all the models. Multicollinearity was assessed via the variance inflation factor (VIF) with a cut-off of 10.

### Ethical consideration

The Ministry of Health (MoH) Ethical Review Committee, under its Institutional Review Board (IRB), determined that this study did not require additional formal ethics approval because it was based on de-identified secondary data collected under an already ethically approved program. The data were obtained from a feasibility assessment of triple testing for HIV, HBV, and syphilis conducted in four regions under the Prevention of Mother-to-Child Transmission (PMTCT) program, which had previously received ethical clearance from the National Institute for Medical Research (NIMR).

Written informed consent was obtained from all study participants at the time of data collection and adolescents between 15–17 yrs. written informed consent was obtained from their parents/guardians and assent was sought for their participation. Since the data were routinely collected as part of antenatal healthcare services and were fully de-identified, no additional risks were posed to participants. The study adhered to the ethical principles outlined in the Declaration of Helsinki, and all data were handled in accordance with standard ethical guidelines.

## Results

### Sociodemographic characteristics of the study participants

A total of 7,828 pregnant women were included in our study. Approximately half of the pregnant women were aged 25–49 years. (56.0%), with a median age of 26 yrs. and an interquartile range of 21–30 yrs. The majority of them resided in the Mbeya region (57.8%), were married/cohabiting (91.7%), and they had a primary level of education (57.1%). Most of the women were self-employed (80.2%), and they were attended at a health centre (34.9%) (Table 1).

**Table 1:** Sociodemographic characteristics of pregnant women receiving antenatal care in Tanzania.

Variable	Frequency	Percentage
**Age group**		
15-24	3,443	44.0
25-49	4,385	56.0
**Place of residence**		
Dar es salaam	1,324	16.9
Geita	1,300	16.6
Mbeya	4,522	57.8
Njombe	682	8.7
**Marital status**		
Single/divorced/widowed	651	8.3
Married/cohabiting	7,177	91.7
**Level of education**		
No formal education	345	4.4
Primary	4,471	57.1
Secondary	2,689	34.4
Above Secondary	323	4.1
**Employment status**		
Employed	380	4.9
self-employed	6,275	80.2
unemployed	1,173	14.9
**Health facility level**		
Dispensary	2,605	33.3
Health centre	2,733	34.9
Hospital	2,490	31.8

### Prevalence of HIV, HBV and syphilis coinfections among pregnant women in Tanzania

**Fig 2:**
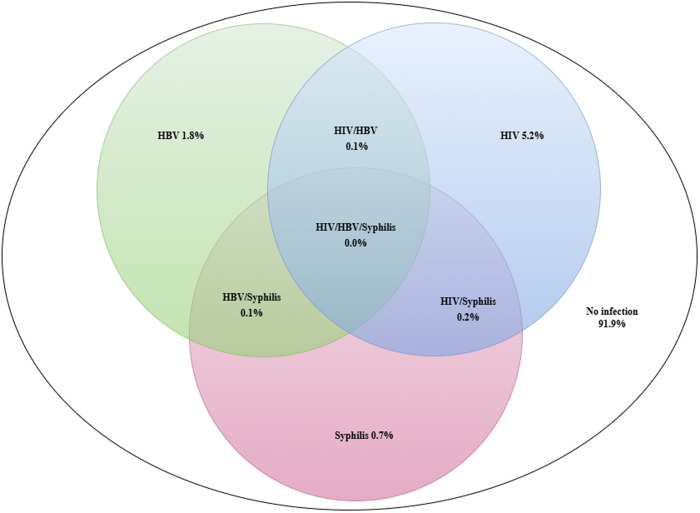
Prevalence of HIV, HBV and syphilis coinfections among pregnant women in Tanzania.

The prevalence rates of HIV, HBV and syphilis were 5.2% (95% CI 4.7–5.7), 1.8% (95% CI 1.5–2.1) and 0.7% (95% CI 0.5–0.9), respectively. The prevalence rates for HIV/HBV, HIV/syphilis, HBV/syphilis and HIV/HBV/syphilis coinfections were 0.1% (95% CI 0.1–0.2), 0.2% (95% CI 0.1–0.4), 0.1% (95% CI 0.0–0.2) and 0.0% (95% CI 0.0–0.1), respectively. There were 32 (0.4%) (95% CI 0.3–0.6) pregnant women with more than one infection (Fig 2).

### Factors associated with HIV/HBV coinfection among pregnant women

From the bivariate analysis, three variables were entered into the multivariate analysis: having multiple sexual partners, having a history of blood transfusion and having a history of tooth extraction. According to multivariate analysis, pregnant women who had more than one sexual partner in the past 6 months were 6.1 times more likely to have HIV/HBV coinfection than women who had one sexual partner (AOR 6.1; 95% CI: 1.3–29.7, P = 0.025) (Table 2).

**Table 2:** Regression analysis for factors associated with HIV/HBV coinfection among pregnant women in Tanzania.

variable	HIV/HBV		Bivariate Analysis		Multivariate Analysis	
	Positive n (%)	Negative n (%)	COR (95%CI)	P value	AOR (95%CI)	P value
**Age group**						
15 - 24	2(0.1)	3,441(99.9)	1			
25 - 49	7(0.2)	4,378(99.8)	2.8(0.6-13.3)	0.207		
**Level of education**						
No formal education	1(0.3)	344(99.7)	1			
Primary	5(0.1)	4,466(99.9)	0.4(0.0-3.3)	0.384		
Secondary	2(0.1)	2,687(99.9)	0.3(0.0-2.8)	0.266		
Tertiary	1(0.3)	322(99.7)	1.1(0.1-17.2)	0.963		
**Place of residence**						
Dar es salaam	1(0.1)	1,323(99.9)	1			
Geita	3(0.2)	1,297(99.8)	3.1(0.3-29.5)	0.333		
Mbeya	4(0.1)	4,518(99.9)	1.2(0.1-10.5)	0.888		
Njombe	1(0.2)	681(99.8)	1.9(0.1-31.1)	0.639		
**Multiple sexual partners**						
≤1	7(0.1)	7,466(99.9)	1			
>1	2(0.6)	353(99.4)	6.0(1.3-29.2)	0.025	6.1(1.3-29.7)	0.025
**Bood transfusion history**						
No	8(0.1)	7,654(99.9)	1			
Yes	1(0.6)	165(99.4)	5.8(0.7-46.6)	0.098	4.9(0.6-42.0)	0.146
**Tooth extraction history**						
No	7(0.1)	7,210(99.9)	1			
Yes	2(0.3)	609(99.7)	3.4(0.7-16.3)	0.129	2.8(0.6-13.9)	0.217
**History of ear pierced**						
No	6(0.1)	5,984(99.9)	1			
Yes	3(0.2)	1,835(99.8)	1.6(0.4-6.5)	0.49		

### Factors associated with HIV/syphilis coinfection among pregnant women

A total of five variables from the bivariate analysis were entered into the multivariate analysis, which included age group, marital status, multiple sexual partners, condom use and tooth extraction history. Pregnant women aged 25–49 years were 13.5 times more likely to have HIV/syphilis coinfection than those aged 15–24 years (AOR 13.5; 95% CI 1.8–103.8, P = 0.012). Compared with single/widowed/divorced pregnant women, married/cohabiting pregnant women had approximately 80% lower odds of having HIV/syphilis coinfection (AOR 0.2; 95% CI 0.1–0.8, P = 0.018) (Table 3).

**Table 3:** Regression analysis for factors associated with HIV/Syphilis coinfection among pregnant women in Tanzania.

Variables	HIV/Syphilis		Bivariate Analysis		Multivariate Analysis	
	Positive n (%)	Negative n (%)	COR (95%CI)	P value	AOR (95%CI)	P value
**Age group**						
15-24	1(0.0)	3,442(100.0)	1			
25-49	16(0.4)	4,369(99.6)	12.6(1.7-95.1)	0.014	13.5(1.8-103.8)	0.012
**Marital Status**						
Single/Divorced/widowed	4(0.6)	647(99.4)	1			
Married/cohabitating	13(0.2)	7,164(99.8)	0.3(0.1-0.9)	0.032	0.2(0.1-0.8)	0.018
**Employment Status**						
Employed	1(0.3)	379(99.7)	1			
Self employed	14(0.2)	6,261(99.8)	0.9(0.1-6.5)	0.873		
Unemployed	2(0.2)	1,171(99.8)	0.7(0.1-7.2)	0.723		
**Multiple sexual Partners**						
≤1	15(0.2)	7,458(99.8)	1			
>1	2(0.6)	353(99.4)	2.8(0.6-12.4)	0.170	1.8(0.4-8.4)	0.483
**Condom use (n = 7,313)** ^**a**^						
Always	1(1.0)	102(99.0)	1			
Never/sometimes	15(0.2)	7,195(99.8)	0.2(0.0-1.6)	0.136	0.2(0.0-1.8)	0.162
**Blood transfusion history**						
No	16(0.2)	7,646(99.8)	1			
Yes	1(0.6)	165(99.4)	2.9(0.4-22.0)	0.304		
**Operation history**						
No	16(0.2)	7,348(99.8)	1			
Yes	1(0.2)	463(99.8)	1.0(0.1-7.5)	0.994		
**Tooth extraction history**						
No	14(0.2)	7,203(99.8)	1			
Yes	3(0.5)	608(99.5)	2.5(0.7-8.9)	0.144	2.0(0.6-7.1)	0.288
**History of ear pierced**						
No	11(0.2)	5,979(99.8)	1			
Yes	6(0.3)	1,832(99.7)	1.8(0.7-4.8)	0.256		

^a^ Data on condom use were available for 7,313 participants. The question was optional in the data collection tool, and 515 participants did not respond.

### Factors associated with HBV/syphilis coinfection among pregnant women

Two variables from the bivariate analysis were entered into the multivariate analysis, namely, marital status and having multiple sexual partners. Multivariate analysis revealed that compared with single/widowed/divorced pregnant women, married/cohabiting pregnant women had approximately 90% reduced odds of having HBV/syphilis coinfection (AOR 0.1; 95% CI 0.0–0.9, P = 0.036). Pregnant women who had more than one sexual partner in the past six months were 16.8 times more likely to have HBV/syphilis coinfection than those who had one sexual partner were (AOR 16.8; 95% CI 2.5–114.9, P = 0.004) (Table 4).

**Table 4:** Regression analysis for factors associated with HBV/Syphilis coinfection among pregnant women in Tanzania.

Variables	HBV/Syphilis		Bivariate Analysis		Multivariate Analysis	
	Positive n (%)	Negative n (%)	COR (95%CI)	P value	AOR (95%CI)	P value
**Marital status**						
Single/widowed/divorced	3(0.5)	648(99.5)	1			
Married/cohabiting	2(0.0)	7,175(100.0)	0.1(0.0-0.4)	0.002	0.1(0.0-0.9)	0.036
**Multiple sexual partners**						
≤1	2(0.0)	7,471(100.0)	1			
>1	3(0.8)	352(99.2)	31.8(5.3-191.1)	<0.001	16.8(2.5-114.9)	0.004
**Tooth extraction history**						
No	4(0.1)	7,213(99.9)	1			
Yes	1(0.2)	610(99.8)	3.0(0.3-26.5)	0.333		
**History of ear pierced**						
No	3(0.1)	5,987(99.9)	1			
Yes	2(0.1)	1,836(99.9)	2.2(0.4-13.0)	0.395		

## Discussion

This study estimated the prevalence and associated factors for HIV, HBV and syphilis coinfections among pregnant women in Tanzania. The prevalence rates for HIV/HBV, HIV/syphilis, HBV/syphilis and HIV/HBV/syphilis coinfections were 0.1%, 0.2%, 0.1% and 0.0%, respectively. The factor that was independently associated with HIV/HBV coinfection was having multiple sexual partners. HIV/syphilis coinfection was significantly more common among pregnant women aged 25–49 years. and less commonly among married or cohabiting women. HBV/syphilis coinfection was significantly less common among married/cohabiting women, whereas it was more common among pregnant women with multiple sexual partners.

The prevalence of HIV/HBV coinfection in our study was lower than that reported in studies conducted in Rwanda [12], Ethiopia [14] and Angola [13]. This can be explained by the fact that the prevalence of HIV and HBV infections among pregnant women in Tanzania is lower than that in Ethiopia, Rwanda and Angola. Furthermore, the prevalence of HIV/HBV coinfection in our study is lower than that in the study conducted in Mtwara, Tanzania [15], which is attributed to the fact that the study conducted in Mtwara included women who were known to be HIV positive; hence, this could have increased the risk of HBV infection and hence high HIV/HBV coinfection in that study.

Pregnant women who had multiple sexual partners in the past six months had increased odds of having HIV/HBV coinfection, which is similar to the findings of studies conducted in Adis Ababa, Ethiopia [21], and southern Ethiopia [22]. This is because HIV and HBV are sexually transmitted diseases; therefore, their risk for transmission increases with increasing number of sexual encounters with different people.

The proportion of pregnant women with HIV/syphilis coinfection in our study was lower than that reported in studies conducted in Ethiopia (1.3%) [14], the Republic of Congo (0.7%) [17], Angola (5%) [18] and Botswana (37.7%) [19]. The difference is because of differences in the study population; the study in Botswana included women who were syphilis positive, which could have increased their risk of acquiring HIV. Furthermore, differences in coinfection prevalence could be linked to differences in sociodemographic characteristics, sample size and exposure to risk behaviours across countries.

Pregnant women aged 25–49 years had greater odds of having HIV/syphilis coinfection than those who were younger. This finding is consistent with a study conducted in Rwanda, which revealed that women aged 25 years and older were more likely to have HIV/syphilis coinfection [23]. This is because women in this group are of child-bearing age; hence, they tend to have more frequent coital with unprotected sex with the purpose of having children than younger groups do. Hence, the risk of HIV/syphilis coinfection increases.

Compared with those who were single, divorced or widowed, married and cohabiting women had lower odds of having HIV/syphilis coinfection; this finding is corroborated by a study performed in Turkey, which showed that being single was associated with having HIV/syphilis coinfection [24]. Those who are single or lack a partner tend to have multiple sexual partners, which increases their risk of having HIV/syphilis coinfection, or they tend to be in a relationship with a partner who has multiple partners.

The prevalence of HBV/syphilis coinfection among pregnant women in our study was lower than that reported in studies conducted in southern Ethiopia (3.1%) [25] and northern Ethiopia (0.8%) [14]. The lower prevalence of HBV/syphilis coinfection in our study can be explained by the fact that the prevalence of HBV and syphilis among pregnant women in our study was lower than that reported in studies in Ethiopia. Furthermore, the variation can be explained by disparities in access to and utilization of antenatal care services. For instance, Tanzania has a higher antenatal care coverage, with 94% of pregnant women attending at least one ANC visit and 62% completing four or more visits (TDHS 2022) [26], compared to 74% for first visits and 43% for four or more visits in Ethiopia (EDHS 2019) [27]. Greater ANC coverage in Tanzania may contribute to earlier detection, management, and possible prevention of maternal infections, which could partly explain the lower coinfection rate observed in our study.

Married and cohabiting women had lower odds of having HBV/syphilis coinfection because married/cohabiting individuals are less likely to be infected with HBV [28] and syphilis [17] than single/widowed/divorced individuals are. Unlike married individuals, those who are single are more likely to engage in sexual intercourse with multiple partners, increasing their chances of contracting sexually transmitted infections (STIs).

Pregnant women with multiple sexual partners are more likely to have HBV/syphilis coinfection because having multiple sexual partners is an independent risk factor for the transmission of STIs, which play a significant role in the transmission of HBV [29–32] and syphilis infection [33] since they all share a similar route of transmission.

The prevalence of HIV/HBV/syphilis coinfection in our study was lower than that reported in a study conducted at West China Hospital (3.2%) [34], but it was higher than that reported in a study conducted in Ethiopia, where there were no cases of triple coinfection [14]. This finding highlights the importance of integrating HBV screening with existing HIV and syphilis screening of pregnant women since there are women who are infected by triple coinfections, and their management is crucial in preventing vertical transmission to newborns.

Although the prevalence of coinfections in our study is low, this study provides meaningful and updated evidence from Tanzania, where current data on maternal coinfection rates remain limited. These findings suggests that existing prevention and screening strategies are having a positive impact. However, the detection of any coinfection even at low levels highlights the continued need for routine screening of HIV, HBV and syphilis during antenatal care, given the serious risk of vertical transmission.

Furthermore, understanding the factors associated with HIV, HBV and syphilis coinfections is crucial in the development of targeted interventions aimed at the identification and management of coinfections to prevent vertical transmission. This will help eliminate coinfections through evidence-based approaches.

Despite the fact that our study included a large sample size of participants from four regions in Tanzania, making the findings representative and generalizable, the study was limited by self-reported variables such as the number of sexual partners and condom use, which could have resulted in participants reporting socially desirable answers, hence biasing our results.

## Conclusion and recommendations

HIV, HBV and syphilis coinfections are present among pregnant women in Tanzania. The factor that was independently associated with HIV/HBV coinfection was having multiple sexual partners. HIV/syphilis coinfection was associated with pregnant women aged 25–49 years and marital status. HBV/syphilis coinfection was independently associated with marital status and having multiple sexual partners. Integrating HBV screening with preexisting HIV and syphilis programs is crucial because of the presence of coinfections; furthermore, targeted interventions should prioritize single, child bearing women with multiple sexual partners.

## Supporting information

S1 File Anonymized dataset for analysis. (ZIP)
